# Use of Tetanus Toxoid, Reduced Diphtheria Toxoid, and Acellular Pertussis Vaccines: Updated Recommendations of the Advisory Committee on Immunization Practices — United States, 2019

**DOI:** 10.15585/mmwr.mm6903a5

**Published:** 2020-01-24

**Authors:** Fiona P. Havers, Pedro L. Moro, Paul Hunter, Susan Hariri, Henry Bernstein

**Affiliations:** ^1^Division of Bacterial Diseases, National Center for Immunization and Respiratory Diseases, CDC; ^2^Division Of Healthcare Quality Promotion, National Center for Emerging and Zoonotic Infectious Diseases, CDC; ^3^Department of Family Medicine and Community Health, University of Wisconsin School of Medicine and Public Health, Madison, Wisconsin; ^4^Zucker School of Medicine at Hofstra/Northwell Cohen Children’s Medical Center, New Hyde Park, New York.

## Introduction

Since 2005, a single dose of tetanus toxoid, reduced diphtheria toxoid, and acellular pertussis (Tdap) vaccine has been recommended by the Advisory Committee on Immunization Practices (ACIP) for adolescents and adults (*1*,*2*). After receipt of Tdap, booster doses of tetanus and diphtheria toxoids (Td) vaccine are recommended every 10 years or when indicated for wound management. During the October 2019 meeting of ACIP, the organization updated its recommendations to allow use of either Td or Tdap where previously only Td was recommended. These situations include decennial Td booster doses, tetanus prophylaxis when indicated for wound management in persons who had previously received Tdap, and for multiple doses in the catch-up immunization schedule for persons aged ≥7 years with incomplete or unknown vaccination history. Allowing either Tdap or Td to be used in situations where Td only was previously recommended increases provider point-of-care flexibility. This report updates ACIP recommendations and guidance regarding the use of Tdap vaccines (*3*).

## Background

Two Tdap vaccines are licensed for use in the United States. Boostrix (GlaxoSmithKline) is approved for a single dose in persons aged ≥10 years; Adacel (Sanofi Pasteur) is approved for persons aged 10–64 years. Since 2005, a single booster dose of Tdap has been recommended for children and adolescents aged 11–18 years and adults aged 19–64 years (*1*,*2*) to increase protection against tetanus, diphtheria, and pertussis. Booster doses of Td have been recommended every 10 years (decennial vaccination) to ensure continued protection against tetanus and diphtheria. These recommendations were expanded to include a single dose of Tdap for adults aged ≥65 years in 2012 (although only one Tdap product is approved for use in persons aged ≥65 years, either vaccine administered to a person aged ≥65 years is considered valid) (*4*). Pregnant women are recommended to receive a dose of Tdap during each pregnancy to prevent pertussis in infants too young for routine vaccination (off-label use*) (*3*). If a tetanus toxoid–containing vaccine is indicated for wound management, Td has been recommended for nonpregnant persons aged ≥7 years who had previously received Tdap. For pregnant women, Tdap is recommended in this setting. For previously unvaccinated persons aged ≥7 years, a 3-dose catch-up immunization schedule included only 1 dose of Tdap, preferably as the first dose in the series (off-label use in children aged 7–9 years), and 2 subsequent Td doses at specified intervals (*5*). No further doses of Tdap were routinely recommended, with two exceptions: pregnant women should receive Tdap during each pregnancy (off-label use), and children aged 7–10 years who received Tdap as part of the catch-up schedule were recommended to receive the routine adolescent Tdap booster dose at age 11–12 years (*1*,*2*). In 2010, ACIP evaluated the safety of administering Tdap at intervals <5 years after Td administration (*6*,*7*) and recommended that the dose of Tdap, when indicated, should not be delayed and should be administered regardless of the interval since the last tetanus or diphtheria toxoid–containing vaccine.

In 2013, ACIP reviewed the most recent safety and immunogenicity data available at that time to inform their recommendations regarding a second routine dose of Tdap. ACIP concluded that a second dose of Tdap would be safe and immunogenic at 5- or 10-year intervals (*8*–*12*). However, antipertussis antibodies decline rapidly after the first year (*10*,*13*–*20*), and vaccine effectiveness studies indicated that pertussis protection begins to wane within 2–4 years after receipt of a single Tdap dose (*21*–*23*). This likely limits the impact of a second dose of Tdap on the overall burden of pertussis in the United States (*24*). In addition, Tdap vaccines have an uncertain role in prevention of transmission and in herd protection (*25*,*26*). ACIP concluded that the data did not support a general recommendation for a routine second dose of Tdap, given the likely limited public health impact (*27*).

In January 2019, FDA approved Adacel for a second Tdap dose if administered ≥8 years after the first Tdap dose and for use for tetanus prophylaxis when indicated for wound management if ≥5 years have elapsed since the previous receipt of any tetanus toxoid–containing vaccine (*28*). In light of the new indication for a second dose of Adacel and evidence of Tdap being used frequently in place of Td (*29*), ACIP reassessed current Tdap recommendations. In October 2019, ACIP recommended that either Tdap or Td vaccines could be used in situations where only Td vaccine had been recommended previously. This report provides recommendations for the use of Td or Tdap for the decennial Td booster, tetanus prophylaxis when indicated for wound management, and catch-up immunization schedule for persons aged ≥7 years with incomplete or unknown vaccination history.

## Methods

Beginning in September 2018, the ACIP Pertussis Vaccines Work Group participated in monthly telephone conferences to review Tdap vaccination recommendations. A search of clinical trials published during January 2013–June 2019 that examined Tdap vaccination in adolescents and adults who had previously received Tdap was performed, so the work group could review data that had not previously been reviewed by ACIP. Because of limited data on the use of >1 Tdap dose in the catch-up immunization schedule, the work group also considered published and unpublished safety data on receipt of >1 Tdap dose within a 12-month period in both pregnant women and nonpregnant adolescents and adults. Data from public sector orders (CDC, unpublished data, 2019), commercial insurance claims (Truven Health Analytics, unpublished data, 2019), and a published study from the Vaccine Safety Datalink (VSD) (*29*) were analyzed to assess stakeholders’ values attributed to perceived benefits and harms, acceptability, and implementation considerations regarding use of Tdap in place of Td.

Summaries of evidence, including the evidence to recommendations framework (https://www.cdc.gov/vaccines/acip/recs/grade/tdap-etr.html) and assessment of programmatic considerations, were presented to ACIP at the October 2018, June 2019, and October 2019 meetings. Proposed recommendations were presented to the committee at the October 2019 meeting, and, after a public comment period, were approved by the voting members as follows: either Td or Tdap should be allowed for use in situations where only Td is currently recommended for the decennial Td booster, tetanus prophylaxis for wound management, and catch-up vaccination, including in pregnant women (14 voted in favor, and none opposed).

## Summary of Key Findings

**Safety and immunogenicity.** Two clinical trials found no increased risk for adverse events among adults who received Tdap, compared with those who received Td 10 years after receipt of the initial Tdap dose (*30*,*31*). In addition, the proportion of persons with seroprotective levels of antibodies to tetanus and diphtheria was similar in the Tdap and Td groups. Another clinical trial compared adults receiving a second dose of Tdap 9 years after their initial Tdap dose with adults receiving Tdap for the first time as a control group (*32*). Solicited adverse events, the most frequent of which were injection site pain, fatigue, and headache, were higher in the groups receiving a second dose of Tdap. Grade 3 adverse events, defined in this study as redness and swelling with diameter >50 mm, pain, headaches, fatigue, gastrointestinal symptoms preventing normal activity, and fever with temperature >104°F(40°C), were similar in both groups. A retrospective VSD study identified 68,915 adolescents and adults who had received an initial dose of Tdap and then received another Td-containing vaccine, either a second Tdap (61,394, 89%) or Td (7,521, 11%). There was no statistically significant increase in medical visits for cellulitis, limb swelling, pain in limb, seizure, cranial nerve disorders, paralytic syndromes, encephalopathy, encephalitis, or meningitis among those who received a subsequent dose of Tdap compared with those who received Td (*29*).

Data on the use of >1 Tdap dose in the catch-up immunization schedule are limited. One double-blind, randomized controlled clinical trial enrolled 460 adults aged ≥40 years who had not received a diphtheria or tetanus vaccination for ≥20 years or who had an unknown vaccination history. Subjects were randomized to receive either 3 doses of a Tdap formulation; 1 Tdap-inactivated polio vaccine combination dose, which is not licensed in the United States, followed by 2 Td doses; or 3 Td doses at 0, 1, and 6 months. There was no significant difference in adverse events for subjects receiving 3 Tdap doses, compared with those receiving 3 Td doses, and no significant differences in diphtheria and tetanus seroprotection rates among the three groups (*33*). An analysis of data collected as part of a published VSD retrospective study (*29*) identified 13,599 persons who had received an initial dose of Tdap and then received another Td-containing vaccine within 12 months of the previous Tdap dose, either a second Tdap (11,687, 86%) or Td (1,912, 16%). There was no elevated risk for medical visits for adverse events among those who received a subsequent dose of Tdap compared with those who received Td (CDC/VSD, unpublished data, 2019). Among 34,804 reports to the Vaccine Adverse Event Reporting System (VAERS) (*34*) following receipt of Tdap in nonpregnant and pregnant persons of all ages during January 1, 1990–June 30, 2019, 88 (0.3%) persons had received multiple Tdap doses spaced ≤12 months apart. Among this small group of reports, 21 (24%) were associated with adverse events, the most frequent of which was injection site reactions (8, 38%) (CDC, unpublished data, 2019).

There are no published data comparing rates of adverse events among pregnant women who received multiple doses of Tdap during a single pregnancy with those who received a single Tdap dose and additional Td doses for catch-up vaccination. A cohort study examining reactogenicity of Tdap in pregnant women included only eight study participants who received >1 Tdap dose within a 12-month period; none experienced severe reactions or fever (*35*). A VSD study examining safety of Tdap during pregnancy identified 187 women who had received >1 Tdap dose during a single pregnancy among 633,542 singleton pregnancies screened for potential study inclusion (*36*). Although these 187 women were excluded from the published study, the authors found similar rates of adverse birth outcomes (i.e., small for gestational age, preterm delivery, and low birthweight) in these women compared with women who had received a single Tdap dose in pregnancy (29,155). Among these 187 women who received >1 Tdap dose during pregnancy, one had a medically attended acute adverse event, which was diagnosed as limb pain and swelling 7 days after vaccination. One woman received 3 Tdap doses during a single pregnancy; she did not experience any adverse events, and her baby was born at term (CDC, unpublished data, 2019).

**Acceptability to patients and providers.** Analysis of commercial insurance claims indicated that Tdap claims were 12 times as high as Td claims in adults aged 19–64 years during 2017 (Truven Health Analytics, unpublished data, 2019). In the same year, there were approximately 10 times the number of Tdap doses (441,075) as Td doses (41,881) ordered by providers for adults as public sector purchases (CDC, unpublished data, 2019). These data, in addition to the one published VSD study (*29*) documented that Tdap was widely used in place of Td by clinicians in the United States and suggested acceptability to both patients and health care providers.

**Health impact and economic considerations.** Tdap costs more than Td (*37*). The population-level effectiveness and economic impact of replacing Td with Tdap has been modeled and previously reviewed by ACIP (*24*). However, this analysis and an updated model of the economic impact of substituting Tdap for the decennial Td booster demonstrated that estimates of cost effectiveness are dependent on values for parameters with a high degree of uncertainty, including pertussis incidence, illness severity, initial vaccine effectiveness, duration of protection, and the impact of Tdap on herd protection (*38*). Coupling such uncertainty with the evidence for notable widespread use of Tdap in place of Td, programmatic issues were the main consideration in the decision-making process.

## Rationale for Recommendations

In 2013, ACIP did not support a general recommendation for a routine second dose of Tdap; the rationale was described in previously published guidance (*3*). In 2019, ACIP again concluded that in light of the higher cost of Tdap relative to Td and uncertainty about the impact that receipt of multiple Tdap doses would have on pertussis control and transmission, there continues to be insufficient evidence to preferentially recommend that Tdap replace Td. However, given the reassuring safety profile and evidence of widespread use of Tdap in place of Td, to allow providers more flexibility, either Tdap or Td was recommended for use in situations when previously only Td was recommended. ACIP recommends that either Td or Tdap be used for the decennial Td booster, tetanus prophylaxis for wound management, and for additional required doses in the catch-up immunization schedule if a person has received at least 1 Tdap dose.

## General Recommendations

**Persons aged 11–18 years.** These persons should receive a single dose of Tdap, preferably at a preventive care visit at age 11–12 years. To ensure continued protection against tetanus and diphtheria, 1 booster dose of either Td or Tdap should be administered every 10 years throughout life.

**Persons aged ≥19 years.** Regardless of the interval since their last tetanus or diphtheria toxoid–containing vaccine, persons aged ≥19 years who have never received a dose of Tdap should receive 1 dose of Tdap. To ensure continued protection against tetanus and diphtheria, booster doses of either Td or Tdap should be administered every 10 years throughout life.

**Pregnant women.** No change has been made to the recommendations for routine Tdap immunization during pregnancy. Pregnant women should receive 1 dose of Tdap during each pregnancy, irrespective of their history of receiving the vaccine. Tdap should be administered at 27–36 weeks’ gestation, preferably during the earlier part of this period, although it may be administered at any time during pregnancy (*3*,*5*).

## Tetanus Prophylaxis for Wound Management Recommendations

A tetanus toxoid–containing vaccine is indicated for wound management when >5 years have passed since the last tetanus toxoid–containing vaccine dose. If a tetanus toxoid–containing vaccine is indicated for persons aged ≥11 years, Tdap is preferred for persons who have not previously received Tdap or whose Tdap history is unknown. If a tetanus toxoid–containing vaccine is indicated for a pregnant woman, Tdap should be used. For nonpregnant persons with documentation of previous Tdap vaccination, either Td or Tdap may be used if a tetanus toxoid–containing vaccine is indicated. Complete information on tetanus prophylaxis and the use of tetanus immunoglobulin when indicated for wound management is available at https://www.cdc.gov/mmwr/volumes/67/rr/rr6702a1.htm.

## Catch-Up Immunization Recommendations

**Persons aged 7–18 years.** If persons aged 7–18 years have never been vaccinated against pertussis, tetanus, or diphtheria, these persons should receive a series of three tetanus and diphtheria toxoid–containing vaccines, which includes at least 1 Tdap dose. The preferred schedule is 1 dose of Tdap, followed by 1 dose of either Td or Tdap ≥4 weeks afterward, and 1 dose of either Td or Tdap 6–12 months later. Persons aged 7–18 years who are not fully immunized against tetanus and diphtheria should receive 1 dose of Tdap, preferably as the first dose in the catch-up series; if additional tetanus toxoid–containing doses are required, either Td or Tdap may be used. The vaccination series does not need to be restarted for those with incomplete DTaP history, regardless of the time that has elapsed between doses. The catch-up schedule and minimum intervals between doses are available at https://www.cdc.gov/vaccines/schedules/hcp/child-adolescent.html.

**Persons aged ≥19 years.** If persons aged ≥19 years have never been vaccinated against pertussis, tetanus, or diphtheria, these persons should receive a series of three tetanus and diphtheria toxoid–containing vaccines, which includes at least 1 Tdap dose. The preferred schedule is 1 dose of Tdap, followed by 1 dose of either Td or Tdap at least 4 weeks afterward, and 1 dose of either Td or Tdap 6–12 months later. Persons aged ≥19 years who are not fully immunized against tetanus and diphtheria should receive 1 dose of Tdap, preferably as the first dose in the catch-up series; if additional tetanus toxoid–containing doses are required, either Td or Tdap may be used.

## Prevention of Neonatal and Obstetric Tetanus

Pregnant women who have completed the childhood immunization schedule and were last vaccinated >10 years previously should receive a booster dose of tetanus toxoid–containing vaccine to prevent neonatal tetanus. The risk for neonatal tetanus is minimal if a previously unvaccinated woman has received at least 2 properly spaced doses of a tetanus toxoid–containing vaccine during pregnancy; at least 1 of the doses administered during pregnancy should be Tdap, administered according to published guidance (*3*). If >1 dose is needed, either Td or Tdap may be used. The 3-dose primary series should be completed at the recommended intervals.

## CDC Guidance

**Catch-up immunization.** For persons aged 7–9 years who receive a dose of Tdap as part of the catch-up series, an adolescent Tdap dose should be administered at age 11–12 years. If a Tdap dose is administered at age ≥10 years, the Tdap dose may count as the adolescent Tdap dose.

## Inadvertent Administration

**Persons aged ≥7 years.** DTaP is not indicated for persons aged ≥7 years. If DTaP is administered inadvertently to a fully vaccinated^†^ child aged 7–9 years, an adolescent Tdap dose should be administered at age 11–12 years. If DTaP is administered inadvertently to an undervaccinated child aged 7–9 years, this dose should count as the Tdap dose of the catch-up series, and the child should receive an adolescent Tdap dose at age 11–12 years. If DTaP is administered inadvertently to a person aged ≥10 years, this dose should count as the adolescent Tdap dose routinely administered at age 11–12 years.

**Fully vaccinated** **children aged 7–10 years.** If a fully vaccinated child aged 7–9 years receives Tdap, the Tdap dose should not be counted as valid. The adolescent Tdap dose should be administered as recommended when this child is aged 11–12 years. The preferred age at administration for the adolescent Tdap dose is 11–12 years. However, if Tdap is administered at age 10 years, the Tdap dose may count as the adolescent Tdap dose.

## Off-Label Use of Vaccine

Off-label indications based on age and pregnancy status have not changed (Table). New off-label indications for Adacel would include any additional routine or catch-up Td dose beyond a second dose administered ≥8 years after an initial Tdap dose, if not given for wound prophylaxis within the specified guidance. Any additional doses of Boostrix administered beyond the single licensed dose are considered off-label. The work group did not find any reason to distinguish between these two products in making its recommendations.

**TABLE:** Food and Drug Administration (FDA)–approved and off-label recommendations for licensed tetanus toxoid, reduced diphtheria toxoid, and acellular pertussis (Tdap) products — United States, 2019

Licensed Tdap product	FDA-approved indications for use and administration	Off-label uses		
		Decennial Td booster	Tetanus prophylaxis for wound management	Catch-up immunization,* including during pregnancy^†^
**Adacel** ^§^	Age: 10–64 years	Age: ≥65 years	Age: <10 or ≥65 years	Age: 7–9 years
	Routine booster ≥5 years after a dose of DTaP or Td vaccine, with a second dose ≥8 years after first (any) Tdap dose	Any dose beyond second Adacel dose administered ≥8 years after first Tdap dose		>1 Tdap dose
	Tetanus prophylaxis if ≥5 years have elapsed since the last tetanus-containing vaccine			
**Boostrix** ^§^	Age: ≥10 years	Any dose if previously received Tdap	Age: <10 years	Age: 7–9 years
	Single dose ≥5 years after a dose of DTaP or Td vaccine		Any dose if previously received Tdap	>1 Tdap dose
	Tetanus prophylaxis if no previous Tdap			

**Abbreviations**: DTaP = diphtheria and tetanus toxoids and acellular pertussis vaccine; Td = tetanus and reduced diphtheria toxoid.

* Persons with incomplete or unknown vaccination history should receive a single dose of Tdap, preferably as the first dose of the 3-dose catch-up series; if additional tetanus toxoid–containing doses are needed, either Td or Tdap vaccine may be used.

**^†^** Both Tdap vaccines may be administered during pregnancy with the same intervals and restrictions (vaccine specific) as would apply to a nonpregnant person.

^§^ Package inserts for indications and intervals for wound management are available at https://www.fda.gov/media/119862/download (Adacel) and https://www.fda.gov/media/124002/download (Boostrix).

**Contraindications and precautions.** Contraindications and precautions are unchanged from previous recommendations (*3*).

**Reporting of vaccine adverse reactions.** Adverse events occurring after administration of any vaccine should be reported to VAERS. Reports can be submitted to VAERS online, by fax, or by mail. Additional information about VAERS is available by telephone (1-800-822-7967) or online (https://vaers.hhs.gov).

## Future Research and Monitoring Priorities

ACIP will continue to review data on Td and Tdap as they become available, examine the necessity and frequency of booster doses for protection against tetanus and diphtheria, and consider any needed policy changes. As with all vaccines, CDC will use VAERS and VSD to monitor adverse events following immunization.

**Summary**

**What is already known about this topic?**

Repeat doses of tetanus toxoid, reduced diphtheria toxoid, and acellular pertussis (Tdap) vaccine at 5- and 10-year intervals are safe and immunogenic.

**What is added by this report?**

ACIP recommendations have been updated to allow either tetanus and diphtheria toxoids (Td) vaccine or Tdap to be used for the decennial Td booster, tetanus prophylaxis for wound management, and for additional required doses in the catch-up immunization schedule if a person has received at least 1 Tdap dose.

**What are the implications for public health practice?**

Allowing either Tdap or Td to be used in situations where Td only was previously recommended increases provider point-of-care flexibility.

## Footnotes

* Off-label use is the use of pharmaceutical drugs for an unapproved indication or in an unapproved age group, dosage, or route of administration.

† Fully vaccinated is defined as having received 5 valid doses of DTaP or 4 valid doses of DTaP if the fourth dose was administered on or after the fourth birthday.
